# Development of a Novel Backbone Cyclic Peptide Inhibitor of the Innate Immune TLR/IL1R Signaling Protein MyD88

**DOI:** 10.1038/s41598-018-27773-8

**Published:** 2018-06-21

**Authors:** Shira Dishon, Adi Schumacher, Joseph Fanous, Alaa Talhami, Ibrahim Kassis, Dimitrios Karussis, Chaim Gilon, Amnon Hoffman, Gabriel Nussbaum

**Affiliations:** 1 Institute of Dental Sciences, Hebrew University-Hadassah Faculty of Dental Medicine, Ein Kerem, 91120 Jerusalem, Israel; 2 Institute for Drug Research, School of Pharmacy, Faculty of Medicine, The Hebrew University of Jerusalem, Ein Kerem, 91120 Jerusalem, Israel; 3 Institute of Chemistry, The Hebrew University of Jerusalem, Safra Campus, Givat Ram, Jerusalem, 91904 Israel; 4 Department of Neurology and Laboratory of Neuroimmunology, Hadassah-Hebrew University Medical Center, Ein Kerem, 91120 Jerusalem, Israel

## Abstract

MyD88 is a cytoplasmic adaptor protein that plays a central role in signaling downstream of the TLRs and the IL1R superfamily. We previously demonstrated that MyD88 plays a critical role in EAE, the murine model of multiple sclerosis, and showed that the MyD88 BB-loop decoy peptide RDVLPGT ameliorates EAE. We now designed and screened a library of backbone cyclized peptides based on the linear BB loop peptide, to identify a metabolically stable inhibitor of MyD88 that retains the binding properties of the linear peptide. We identified a novel cyclic peptide protein mimetic that inhibits inflammatory responses to TLR ligands, and NFκB activation in response to IL-1 activation. The inhibitor, *c*(MyD 4-4), is metabolically stable in comparison to the linear peptide, blocks MyD88 in a specific manner, and inhibits MyD88 function by preventing MyD88 dimerization. Finally, treatment of mice with *c*(MyD 4-4) reduced the severity of clinical disease in the murine EAE model of multiple sclerosis. Thus, modulation of MyD88-dependent signaling using *c*(MyD 4-4) is a potential therapeutic strategy to lower innate immune inflammation in autoimmune CNS disease.

## Introduction

The Myeloid differentiation primary response 88 (MYD88) adaptor bridges between receptors of the Toll-like receptor (TLR) and Interleukin 1 (IL-1) receptor (R) families (except TLR3) and their downstream kinases, leading to activation of NFκB and MAPK pathways^1,2^. Therefore, innate immune signaling in response to a diverse set of chemical structures from the microbial world, and endogenous interleukin 1 family members, converges on a single protein, MyD88^3^. It follows that deficiency in MyD88 has profound effects on the innate immune system leading to susceptibility to infection on the one hand^4,5^, and resistance to inflammatory tissue damage, on the other^6–10^. In the mouse experimental autoimmune encephalomyelitis (EAE) model of multiple sclerosis, MyD88 deficiency confers near absolute resistance to the induction of disease by immunization with myelin epitopes or adoptive transfer of encephalitogenic T cells (where the role of adjuvant is less pronounced)^11–14^. Immunization of MyD88-deficient mice with a peptide of myelin oligodendrocyte glycoprotein (MOG_35–55_) mixed in Complete Freund’s Adjuvant (CFA), or adoptive transfer of wild-type anti-MOG T helper (Th)-1/Th17 cells, induces IL-10 producing T cells that down-regulate EAE^12^. Importantly, although humans with MyD88 inactivating mutations do not display the same infectious phenotype as MyD88-knockout mice^15^, we recently demonstrated that MyD88 plays a similar role in human antigen presenting cells (APCs) to that demonstrated in the murine system – in both, inhibition of APC-expressed MyD88 induces a Th1/Th17 to Th2 shift in responding T cells^16^. Therefore, MyD88 is an attractive therapeutic target in autoimmune CNS inflammation.

MyD88 consists of an N terminal death domain (DD), a C-terminal Toll/Interleukin-1 receptor (TIR) domain, and a short connecting intermediary domain (INT)^17^. The TIR domain is composed of five β-strands alternating with five α-helices. Resolution of crystal structures of human TLR1, TLR2 and MyD88 show a functionally important conserved domain among TIR domains in the loop connecting the second β-strand with the second alpha helix termed the “BB loop”^18,19^. The heptameric peptide RDVLPGT is the target site in the BB loop^20^ and three amino acids (Arginine, Aspartic acid and Proline) are highly conserved across all TIR containing proteins (the absence of Proline in TLR3 may explain the MyD88-independent signaling of this receptor)^21^. The MyD88 BB loop governs dimerization of MyD88, an essential step for initiation of oligomerization, myddosome formation, and downstream inflammatory signaling^22,23^. The heptameric BB loop peptide competitively inhibits MyD88 homodimerization^20^ and ameliorates inflammatory disease in several animal models^16,24–27^, suggesting that targeting the BB loop is a feasible way to inhibit MyD88 signaling *in vivo*^28–30^.

Linear peptides are rapidly degraded by intestinal and plasma peptidases and are not orally bioavailable due to metabolizing enzymes in the intestinal lumen^31–33^. Small molecule peptidomimetics are usually metabolically stable, however the naturally selected properties of the peptide that confer target specificity and binding properties can only be approximated^34,35^. We developed a metabolically stable version of the MyD88 decoy peptide via backbone cyclization^36^. Backbone cyclization of peptides takes advantage of the naturally selected structure of the peptide while providing a ring element that decreases conformational freedom and sensitivity to metabolizing enzymes. We show below that the cyclic MyD88 BB loop peptide is more bioactive than the linear peptide and is resistant to degradation in the intestine and plasma. The cyclic peptide inhibitor blocks human and mouse TLR2 and TLR4 stimulation, inhibits MyD88 dimerization, and reduces clinical disease in EAE.

## Results

### Backbone cyclized derivatives of the MyD88 BB loop linear peptide RDVLPGT

**Figure 1:**
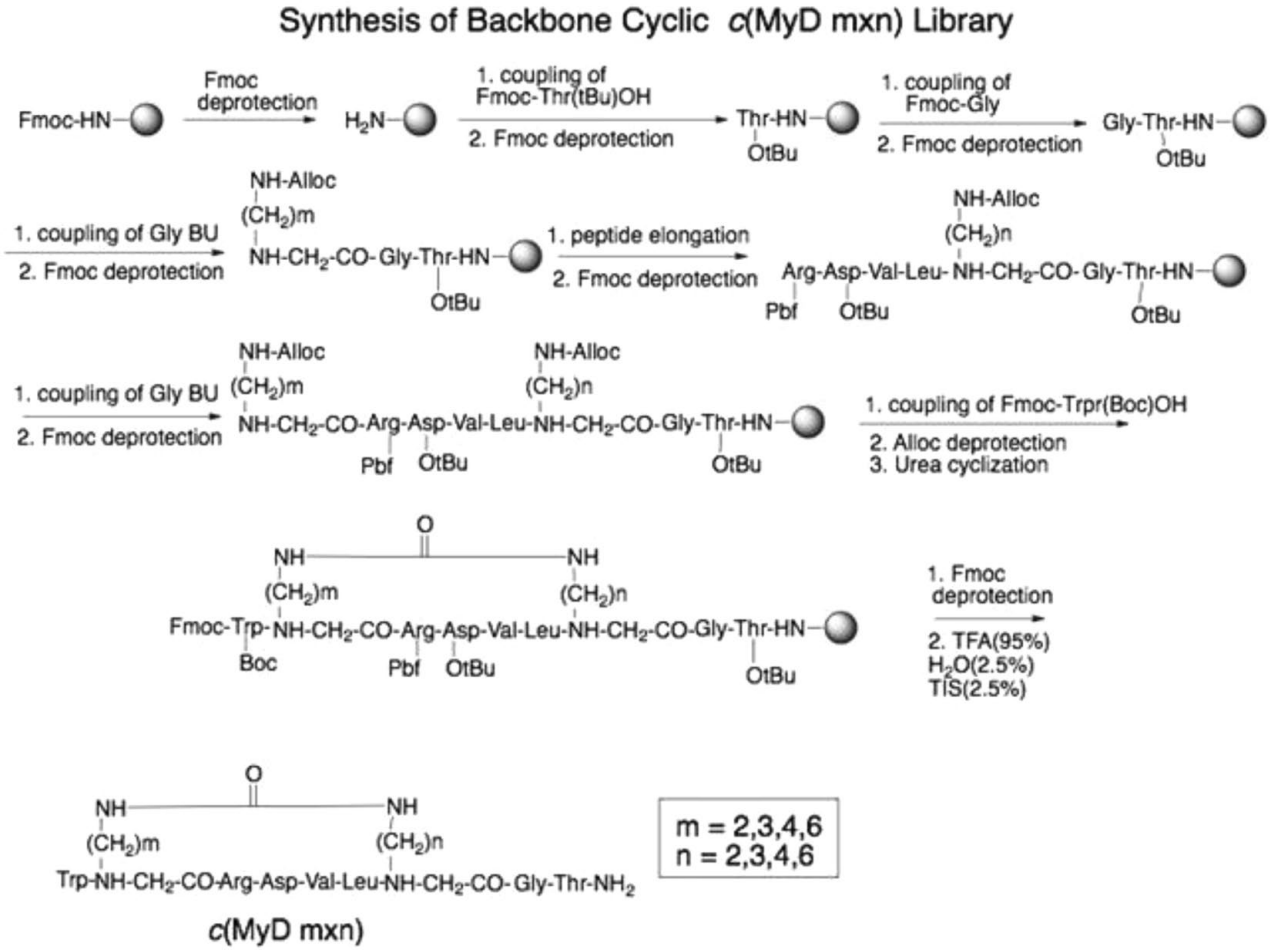
Synthesis of *c*(MyD m-n) backbone cyclic peptide library.

MyD88 function depends on dimerization through the TIR domain^37^. The RDVLPGT peptide corresponds to the region between the βB strand and the αB helix^18^ (the “BB loop”) and competitively inhibits MyD88 dimerization and function^20^. We screened a library of backbone cyclized peptides to identify a metabolically stable derivative of the BB loop peptide that retains the binding and functional properties of the parent peptide. The library was designed using two non-natural building blocks as previously reported^38^. The method, called cycloscan^39^, is based on the concept of screening focused backbone cyclic libraries with spatial diversity that maintain the original side chains of the active region in the protein (RDVLPGT), Backbone cyclization combines N-alkylation with cyclization. In the case of the MyD88 BB-loop decoy peptide RDVLPGT, the tertiary amide bond preceding proline served as one of the anchor sites and was therefore replaced by an N-Alkylated glycine building unit (AGBU) to allow backbone cyclization to the second AGBU at the N-terminus (Fig. 1). Tryptophan was added to the N-terminal AGBU for quantification purposes (Fig. 1). Following the above procedure 16 backbone cyclic peptides were prepared with various ring size and position of the urea bridge in the ring (Table S1). To screen the library, human THP-1 macrophages were pre-treated with the cyclic peptides and then stimulated with Pam3CSK4, a synthetic TLR2/1 lipopeptide agonist. The two compounds that inhibited production of hTNFa strongest, without causing cytotoxicity, were composed of either 4 carbons on each arm (*c*(MyD 4-4)) or 6 carbons on each arm (*c*(MyD6x6)). These compounds were synthesized in larger amounts and re-compared in the same assay (Fig. S1) that demonstrated the superiority of the *c*(MyD 4-4).

### Metabolic stability of *c*(MyD 4-4)

**Figure 2:**
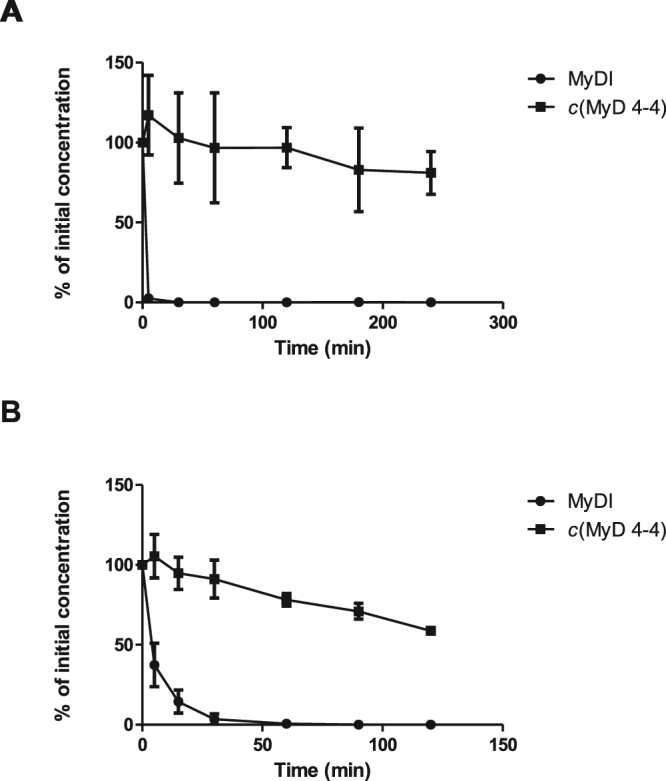
Metabolic stability of linear and cyclic peptides. (**A**) Stability in rat plasma. (**B**) Stability in brush border membrane vesicles (BBMVs). The tested peptides were mixed with fresh plasma (up to 240 minutes) or BBMVs (up to 120 minutes). Peptides were tested in triplicate and data is expressed as the mean ± SEM.

Metabolic instability is one of the major drawbacks of linear peptides^31,32^. Peptide metabolism in the plasma is essential for regulation of important physiological processes of hormones, antibodies and other enzymes^40^. Additionally, peptide metabolizing enzymes in the intestinal lumen degrade dietary proteins to tri/dipeptides and single amino-acids^33^. We compared the stability of the linear RDVLPGT inhibitory peptide (MyDI) to the cyclic *c*(MyD 4-4) (Fig. 2). Both linear and cyclic MyDI peptides were incubated with either rat plasma or brush border membrane vesicles (BBMVs) derived from the lumen of rat intestines^41,42^. The linear peptide was rapidly degraded in the presence of plasma or BBMVs. In contrast, *c*(MyD 4-4) was highly stable in plasma and at 240 minutes >90% of the starting material was detected (Fig. 2A). Similarly, roughly 80% of the *c*(MyD 4-4) was recovered at the end of the 2 h incubation with BBMVs, arguing that cyclization stabilizes the peptide in the harsh enzymatic environment of the intestinal lumen (Fig. 2B).

### *c*(MyD 4-4) blocks human and mouse macrophage TLR2 and TLR4 stimulation

**Figure 3:**
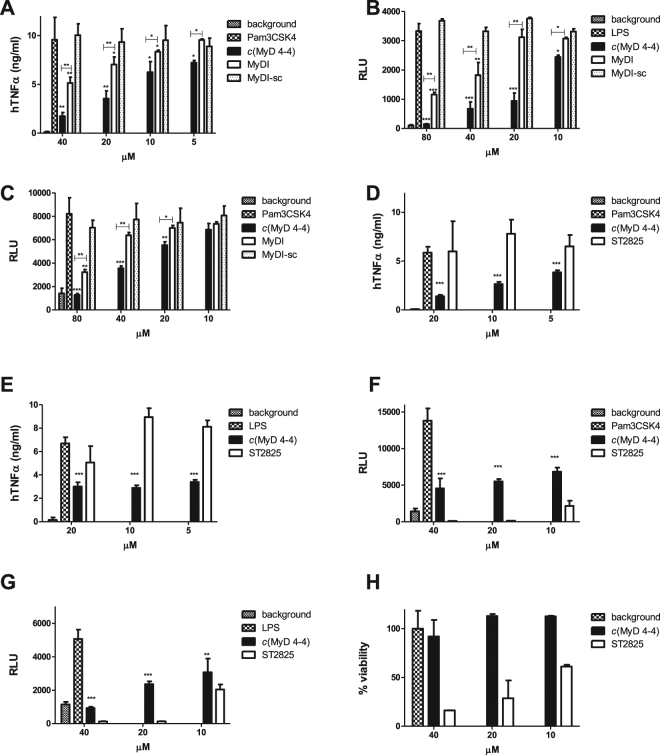
*c*(MyD 4-4) blocks TLR-mediated macrophage activation. (**A**) THP-1 cells were treated with inhibitors for 3 hr prior to activation with the TLR2 ligand Pam3CSK4 200 pg/ml for 24 h. hTNFα levels were determined by ELISA. One representative experiment of three independent experiments is shown. Asterisks represent the significance between treated cells and untreated Pam3CSK4-stimulated control. Asterisks above a cross bar represent the comparison between treatment with *c*(MyD 4-4) and treatment with MyDI. (**B**,**C**) Luminescence levels from the NFκB-luc murine RAW264.7 cells. The different conditions were set according to preliminary assays of each cell line. NFκB-luc RAW264.7 cells were treated with inhibitors for 24 hr and then cells were stimulated with Pam3CSK4 (10 ng/ml) (**C**) or LPS (50 ng/ml) (**D**) for 4 hr and luminescence levels were determined. One representative experiment of three independent experiments is shown. Asterisks alone represent the significance between the cells treated with *c*(MyD 4-4) and the untreated cells stimulated with Pam3CSK4 or LPS. Asterisks above cross bars represent the significance between cells treated with *c*(MyD 4-4) vs. cells treated with MyDI. (**D**–**H**) Treatment with *c*(MyD 4-4) was compared to treatment with ST2825. One representative experiment of three independent experiments is shown. Asterisks represent the significance between cells treated with *c*(MyD 4-4) and the Pam3CSK4 or LPS-stimulated control. (**D**,**E**) THP-1 cells were treated with *c*(MyD 4-4) or ST2825 for 3 hr and then stimulated with the TLR2 ligand Pam3CSK4 or TLR4 ligand LPS. Human (h) TNFα levels were determined by ELISA at 24 hr. (**F**,**G**) NFκB-luc RAW264.7 cells were treated with the indicated compounds for 24 hr and then stimulated with Pam3CSK4 (F) or LPS (**G**) for 4 hr after which luminescence was determined. (**H**) Viability of the NFκB-luc cells treated with *c*(MyD 4-4) vs. ST2825.

We next compared the activity of the *c*(MyD 4-4) compound to the linear BB loop peptide, and to a commercially available small molecule inhibitor of MyD88, ST2825^43^. Incubation with *c*(MyD 4-4) blocked human macrophage cytokine production in response to TLR2 stimulation (Pam3CSK4) significantly more than the linear MyDI peptide (Fig. 3A), and was also more inhibitory than the MyDI peptide when mouse macrophages carrying an NFκB-luciferase reporter gene were activated by Pam3CSK4 or LPS, a TLR4 ligand (Fig. 3B,C). Surprisingly, ST2825 did not block human macrophage cytokine production when macrophages were stimulated with Pam3CSK4 or LPS (Fig. 3D,E). ST2825 was highly cytotoxic to RAW264.7 NFκB-luc cells (Fig. 3F–H), precluding comparison to *c*(MyD 4-4). Thus, *c*(MyD 4-4) is a metabolically stable cyclic peptide inhibitor of TLR2/4 driven inflammatory signaling.

### *c*(MyD 4-4) and the linear MyDI peptide bind to the same region of the MyD88 TIR domain

**Figure 4:**
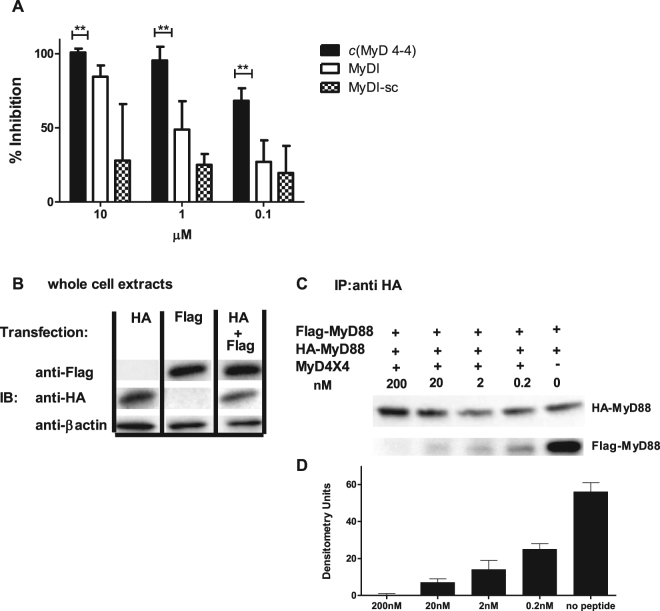
*c*(MyD 4-4) binds to the MyD88 TIR domain and inhibits MyD88 dimerization. (**A**) *c*(MyD 4-4) binds to the MyD88 TIR domain: Recombinant MyD88 TIR protein was attached to a plate and binding of biotinylated linear BB loop peptide was competitively inhibited by the non-biotinylated linear peptide or by *c*(MyD 4-4), but not by the scrambled peptide. The mean of three independent experiments is presented. (**B**,**C**) *c*(MyD 4-4) inhibits MyD88 dimerization: (**B**) WB analysis of HA-MyD88 and Flag-MyD88 expression in singly or doubly-transfected HEK293 cells. Full length gels are presented in Supplementary Figure S3 (**C**) HA-MyD88/Flag-MyD88 co-transfected cells were treated with or without *c*(MyD 4-4) (0.2nM-200 nM) for 3 hr and then stimulated with IL-1β for 30 minutes prior to preparation of cell lysates. Lysates were immunoprecipitated (IP) with anti-HA antibody, and analyzed by anti-HA or anti-Flag WB. (**D**) The extent of dimerization was determined by densitometry. (**B**–**D**) Results are representative of three independent experiments.

The BB loop is a highly conserved region of the MyD88 TIR domain. In particular, the proline at position P200 of MyD88 is highly conserved through evolution and is present in TIR domains of adaptor proteins and all TLRs except TLR3^21^. Since backbone cyclization opened the pyrrolidine ring of the proline side chain, the cyclization could disrupt the structure of the peptide and alter its binding properties compared to the linear peptide. We therefore tested by competition if *c*(MyD 4-4) binds to the same region of the TIR domain as the linear BB loop peptide. To this end, we produced recombinant MyD88 TIR domain (aa 150–296 of the MyD88 protein) as a fusion protein to SUMO^44^ and attached it to ELISA plate wells (Fig. S2A). Biotinylated linear BB loop peptide (Biotin-MyDI) bound the TIR domain in a concentration dependent manner (Fig. S2B) that was inhibited by unlabeled MyDI peptide, but not by a scrambled version of the linear peptide (MyDI-sc) (Fig. 4A). Biotin-MyDI did not bind at any concentration to a control fusion protein consisting of the SUMO protein fused to eGFP (Fig. S2C). Consistent with its binding to the same region of the TIR domain as the linear peptide, *c*(MyD 4-4) competitively inhibited binding of Biotin-MyDI in a concentration dependent manner (Fig. 4A).

### *c*(MyD 4-4) blocks MyD88 dimerization

MyD88 dimerization is mediated through the BB loop region^45,46^ and inhibition of dimerization is considered to be the mechanism by which the MyDI linear peptide blocks TLR signaling. We therefore tested if the *c*(MyD 4-4) has a similar effect on MyD88 dimerization in living cells. To test dimerization, we co-transfected HEK 293 cells with HA-MyD88 and Flag-MyD88. MyD88 dimerization was determined by the detection of Flag-MyD88 following immunoprecipitation of HA-MyD88. Transfection and co-transfection was confirmed by Western Blot analysis (Fig. 4B). 48 hr after co-transfection cells were incubated with different concentrations of *c*(MyD 4-4), and stimulated for 30 min with IL-1β to activate MyD88. Cells were then lysed, and MyD88 was immunoprecipitated using anti-HA antibody followed by immunoblot analysis with anti- Flag antibody. MyD88 homodimerization was strongly inhibited by *c*(MyD 4-4) in a concentration dependent manner (Fig. 4C,D).

### *c*(MyD 4-4) specifically blocks the response to TLR/IL-1 stimulation

**Figure 5:**
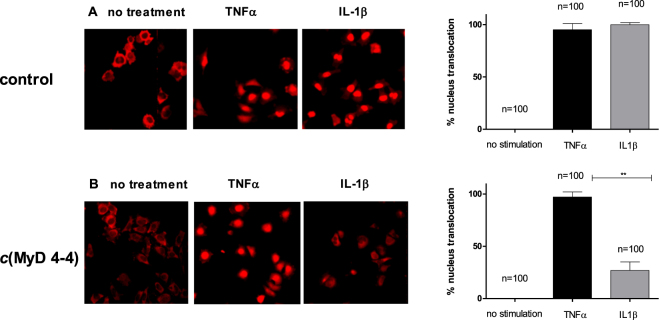
*c*(MyD 4-4) blocks NFκB p65 translocation in response to IL-1β but not in response to TNFα. (**A**,**B**) HeLa cells were stimulated with TNFα or IL-1β, and NFκB localization was determined with anti-p65 conjugated to rhodamine. The bar graphs represent the percent of cells with nuclear localized p65. (**A**) HeLa cells. (**B**) HeLa cells treated for three hr with 20 µM *c*(MyD 4-4). The percentage of cells with nuclear p65 was determined in three independent experiments and the standard deviations between experiments is indicated by the error bars. The number of cells quantified is indicated.

We next confirmed the specificity of *c*(MyD 4-4) inhibition by activating cells with a MyD88-dependent signal, IL-1β, vs. a MyD88-independent signal, TNFa. The ability of *c*(MyD 4-4) to block NFκB activation in these two settings was determined by tracking the translocation of p65 from the cytoplasm to the nucleus in response to each signal. *c*(MyD 4-4) blocked p65 nuclear translocation in response to IL-1β stimulation, but had no effect on NFκB translocation in response to TNFα stimulation (Fig. 5A,B), suggesting that the cyclic compound inhibition is specific, similar to the activity of the linear parent peptide^16^.

### *c*(MyD 4-4) inhibits T cell IFNγ and IL-17 secretion and ameliorates EAE

**Figure 6:**
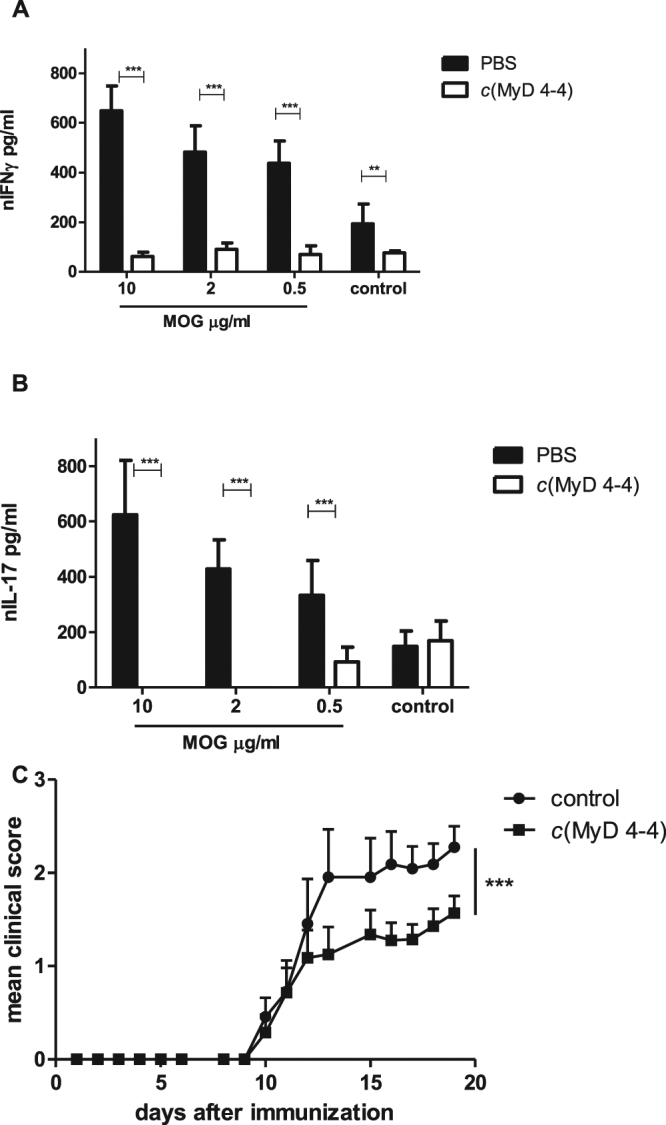
*c*(MyD 4-4) ameliorates EAE. EAE was induced by MOG_35–55_/CFA immunization. Mice were immunized with MOG_35–55_/CFA on day 0, with PTX administration on days 0 and 2. Groups of mice were treated with *c*(MyD 4-4) (4 mg/kg) i.p. or equivalent volume PBS three times a week. (**A**,**B**) Eleven days following EAE induction and treatment with 4 mg/Kg *c*(MyD 4-4) or PBS, DLN were harvested and cells were activated *ex-vivo* with increasing doses of MOG_35-55_ peptide (0.5, 2 and 10 µg/ml). (**A**,**B**) Supernatants were tested for the indicated cytokine after 72 hr of activation, and normalized to the number of cells. Results are representative of two independant experiments, and the 2-Tailed t test was used for statistical evaluation. (**C**) Groups of mice were treated with 4 mg/kg *c*(MyD 4-4) (squares) or PBS (circles) three times a week. The graph shows differences in clinical scores between mice treated with *c*(MyD 4-4) (n = 14 mice per group) and controls (n = 11 mice per group). Data are mean ± s.e.m. and significantly different by two way analysis of variance (ANOVA).

MyD88-dependent signaling plays a major role in autoimmunity^11,14,47,48^, and we recently reported that inhibition of MyD88 can lower disease scores in the EAE mouse model of multiple sclerosis^16^. To investigate the *in vivo* effects of *c*(MyD 4-4) treatment on the EAE disease profile, we immunized mice with MOG_35–55_ peptide emulsified in CFA, and administered Pertussis toxin (PTX) on day zero and at 48 hrs. Mice were treated i.p. three times a week with 4 mg/kg *c*(MyD 4-4) or PBS. To determine the effect of *c*(MyD 4-4) on the developing autoimmune response, we harvested draining lymph nodes (DLN) from immunized mice eleven days following immunization. DLN of treated mice were smaller and contained fewer cells (3 × 10^6^ ± 0.5 × 10^6^) than DLN of control mice (50 × 10^6^ ± 7 × 10^6^). DLN cells from control and *c*(MyD 4-4) treated mice were activated *ex-vivo* with increasing doses of MOG_35-55_, and cytokine secretion was analyzed 72 h after activation and normalized for cell number. As shown in Fig. 6A,B, T cells from DLNs of mice treated with *c*(MyD 4-4) secreted significantly less IFNγ and IL-17 than control treated mice. Finally, groups of treated vs. control mice were followed for EAE disease activity; treatment with *c*(MyD 4-4) led to significantly reduced EAE disease severity (Fig. 6C).

## Discussion

We developed a novel inhibitor of MyD88 directed to the central BB loop region of the TIR domain that is responsible for TIR-TIR interactions. Following MyD88 dimerization mediated by the TIR domain, the MyD88 death domain (DD) interacts with IRAKs through DD-DD interactions leading to assembly of the myddosome complex and activation of inflammatory responses^22^. TIR-TIR interactions are not as stable as DD-DD interactions^49^, suggesting that targeting TIR-TIR binding is a more feasible means of blocking myddosome assembly. The BB loop peptide RDVLPGT is highly conserved and maps to the area of interaction between MyD88 monomers^43^. The linear peptide disrupts MyD88 dimerization and inhibits hyperthermia in response to IL-1 *in vivo*^50^. Blocking MyD88 strongly influences adaptive immunity by inducing a shift away from Th1/Th17 type responses^16^ resulting in protection in several autoimmune/inflammatory disease models^6–9,16^. Despite excellent target specificity, pharmacologic limitations of peptides such as their high sensitivity to proteases restrict their potential as drug candidates. Indeed, we show that the RDVLPGT peptide is rapidly degraded in the presence of plasma or BBMVs. Several groups have identified small molecules that mimic the structural elements and the bioactive conformation of the linear MyDI peptide, either through rational design^43,51^ or screening of molecular libraries^52,53^. These small molecules overcome the pharmacologic limitations of peptides at the cost of losing the naturally selected target binding properties. Peptide backbone cyclization, in contrast, preserves target specificity while conferring resistance to endo and exopeptidases rigidity^54,55^. Backbone cyclization can also improve biological activity by reducing conformational entropy.

*c*(MyD 4-4) was selected from a library of backbone cyclized peptides based on the RDVLPGT BB loop peptide. The bridge is anchored on the nitrogen of the proline residue replacing the pyrrolidine ring. Proline is unique in its cyclic structure among the ribosomally encoded amino acids since it contains a secondary amine formed by the pyrrolidine ring. The peptide bond preceding proline is therefore a tertiary amide bond that is exploited in proteins due to its ability to adopt the cis conformer leading to turns in the protein structure^56^. Furthermore, the conformational influence exerted by proline in peptides is very similar to that of N-alkylated amino acids^57^, and proline containing bioactive peptides can therefore be modified by the synthetic incorporation of N-alkylated amino acids^58^. The MyD88 P200 proline of the BB loop is highly conserved in TIR domains of TLRs and IL-1R family members and serves to confer specificity to TIR-TIR domains as evidenced by the lack of P200 in the TIR domain of TLR3, the only TLR that does not interact with MyD88^21^. Thus, the conserved proline residue in MyD88 BB-loop decoy peptide was replaced by an N-Alkylated glycine building unit that allows backbone cyclization. The creation of a library of backbone cyclized MyD88 inhibitory peptides enabled screening of compounds with spatial diversity that maintained the original side chains of the BB loop peptide.

The *c*(MyD 4-4) peptide is metabolically stable in human plasma and even in the presence of intestinal enzymes (BBMVs), conditions that lead to rapid degradation of the linear peptide. Confirmation that *c*(MyD 4-4) binds to the same site on the MyD88 TIR domain as the linear RDVLPGT peptide was especially important in light of the chemical modification to the conserved P200 side chain. Furthermore, *c*(MyD 4-4) blocks MyD88 function through the same mechanism of action as the linear peptide, i.e. prevention of MyD88 dimerization. Importantly, *c*(MyD 4-4) inhibits MyD88 dimerization at a 10-fold lower concentration compared to the linear peptide. Functionally, *c*(MyD 4-4) blocks human and mouse macrophage activation by TLR2 and TLR4 agonists better than the linear peptide, and better than the commercially available small molecule inhibitor ST2825. Therefore, the backbone cyclized *c*(MyD 4-4) is a metabolically stable inhibitor of MyD88 function with preserved target specificity and improved biological activity.

The phenotype of patients carrying spontaneous MyD88 mutations highlights the dominant role of the TIR domain in controlling myddosome assembly and subsequent inflammatory signaling. To homodimerize, residues in the BB loop of one MyD88 TIR domain interact with the βD and βE strands and the αE helix of the partner TIR domain^21,49^. The R196C polymorphism that affects the Arg of the BB loop (the first Arg of the MyDI peptide), disrupts TIR homodimerization leading to loss of NF-κB signaling and childhood susceptibility to pyogenic bacterial infection^15^. The mutagenesis study of Ohnishi H *et al*.^17^ further demonstrated that R196A reduces MyD88 interaction with MAL, the adaptor protein that bridges between TLR4 and MyD88, leading to NFκB loss of function. The L252P gain of function mutation described in hematological malignancies is located in the βD strand^59^ that interacts with the BB loop, and constitutively activates NF-κB complex activation through formation of spontaneous myddosome clusters^60^. R288A is an additional mutation identified by Ohnishi *et al*.^17^ that decreases NFκB activation via decreased affinity between MyD88 and MAL; R288A is located in the αE strand that mediates BB loop attachment. Taken together, these studies suggest that the BB loop region and the domains that interact with it are ideal targets for calibrating NFκB activation during inflammatory responses.

Innate immune inflammatory signals drive Th1/Th17 mediated autoimmune diseases^47^. In the absence of MyD88, mice are mostly resistant to the induction of autoimmune diseases such as collagen induced arthritis and EAE^14,61^. In fact, MyD88-dependent signaling is required for previously activated encephalitogenic T cells to adoptively transfer EAE^11^, suggesting that MyD88 is necessary not only to prime autoimmune T cells but also to reactivate them. The most obvious requirement for MyD88 is in antigen presenting cells, in order to enable them to respond to environmental signals (such as TLR ligands) and educate T cells toward Th1/Th17 differentiation. However, MyD88 is expressed in many immune and non-immune cell types, and can contribute to autoimmunity at multiple points during disease development. Our finding that *c*(MyD 4-4) inhibits differentiation of autoimmune Th1/Th17 cells and ameliorates disease in the EAE model establishes a proof of concept for the therapeutic potential of MyD88 inhibition in this disease. Further refinement of the pharmacologic properties of this MyD88 inhibitor will enable its testing in multiple conditions where innate immune MyD88-dependent signals promote disease pathogenesis^62^.

## Materials and Methods

### Cell culture and reagents

All cell lines: THP-1, HeLa, HEK 239 T and RAW 264.7 were from the ATCC (VA, USA). RAW 264.7 cells were transduced with Luciferase NF-κB reporter plasmid (Qiagen, Hilden, Germany) and cells stably expressing the plasmid were selected in puromycin (Goldbio.com, MO, USA). Cell lines were grown in RPMI (THP-1, HeLa and HEK 239 T) or DMEM (RAW 264.7) (Sigma, Rehovot, Israel) supplemented with Fetal calf serum- FCS (10%), 4mM L-glutamine, 1 mM sodium pyruvate, penicillin (100 units/ml) and streptomycin (0.1 mg/ml) (Biological Industries, Israel) at 37 °C and 5% CO_2_. Escherichia coli LPS was from Sigma, Pam3CSK4 was from Invitrogen (San Diego, CA, USA) and ST2825 was from Adooq BioScience (CA, USA).

### Peptides

MyD88 inhibitor peptide (MyDI, RDVLPGT), or the scrambled version of the peptide (MyDI-sc, PTDLVRG), were synthesized in the Institute of Chemistry, Hebrew University, Jerusalem, Israel, by standard Fmoc chemistry protocols^38^ using Rink amide methylbenzhydrileamine (MBHA) resin (loading, 0.66 mmol/gr) as the solid support, and purified by HPLC. Peptides below 95% purity were excluded from further examination. Linear and scrambled peptides were dissolved in water for further examination. A library of backbone cyclized peptides based on the RDVLPGT sequence was synthesized using a non-commercial N-Fmoc-[N-(Alloc) x-alkyl] glycine building block (AGBU) in place of the proline residue. AGBU synthesis and cyclization were performed according to previously described procedures^39^. Large scale synthesis of the cyclic peptide *c*(MyD 4-4) for *in vivo* studies was performed at Zhejiang Ontores Biotechnologies Co., Ltd, China. Cyclic peptides were dissolved in DMSO and then further diluted in PBS.

### Cytokine analysis

Cytokines were determined using human/mouse OptEIA sets (BD Biosciences, CA, USA) according to the manufacturer’s instructions.

### Peptide Stability

The linear peptide and *c*(MyD 4-4) (10 µg/ml) were mixed with fresh plasma from male Wistar rats (Harlan, Israel) and incubated at 37 °C for 240 min. Triplicate samples were taken at time 0 and after 5, 30, 60, 120, 180 and 240 min. Rat brush border membrane vesicles (BBMV) were prepared by Ca^2+^ precipitation from the combined duodenum, jejunum, and upper ileum of male rats as described^41,42^. Briefly, intestines were washed with ice cold saline and separated from mucus. The intestinal mucosa was separated from the luminal surface and placed immediately into buffer containing 50 nM KCl and 10 mM Tris–HCl (pH 7.5, 4 °C). The samples were then homogenated (Polytron PT 1200, Kinematica AG, Switzerland) and 10 mM CaCl_2_ was added. The homogenate was placed on a shaker for 30 min at 4 °C and then centrifuged 10,000 g for 10 min. The supernatant was separated and centrifuged at 48,000 g for 30 min and an additional two purification steps were undertaken by suspending the pellet in 300 mM mannitol and 10 mM Hepes/Tris (pH 7.5) and centrifuging at 24,000 g/h. The quality of the BBMV purification was tested using the brush border membrane enzyme markers gamma-glutamyl transpeptidase (GGT), leucine amino peptidase (LAP) and alkaline phosphatase (Sigma-Aldrich, St Louis, MO). Peptides were mixed with purified BBMVs in MES buffer (2-(N-morpholino)ethanesulfonic acid, 50 mM pH 7.4) and incubated at 37 °C for 120 min. Triplicate samples were taken at time 0 and after 15, 30, 45, 60, 90, and 120 min.

To determine the concentration of the compounds, experimental samples were diluted 1:1 with ice-cold acetonitrile and centrifuged (11000 RPM, 10 min). The supernatant was separated and evaporated (Vacuum Evaporation System, Labconco, Kansas City, MO). Samples were reconstituted with acetonitrile: water 70:30 and then centrifuged (11000 RPM, 10 min). The amount of the compounds was determined using high performance liquid chromatography mass spectrometry (HPLC-MS) Waters 2695 Separation Module, equipped with Micromass ZQ detector. The resulting solution (100 μl) was injected into the HPLC system. The system was conditioned as follows: Kinetex® 2.6 µm HILIC 100 A, 100 × 2.1 mm column (Phenomenex®, Torrance, CA), an isocratic mobile phase, acetonitrile:water:ammonium acetate buffer (70:10:20 v/v/v), flow rate of 0.2 mL/min at 25 °C.

### Luciferase Assay

Luciferase activity was measured with a Bright Glo™ luciferase assay system (Promega, Madison, WI, USA), in an Infinite 200 Pro plate reader (Tecan, Männedorf, Switzerland), and data are shown as relative luminescence units (RLU).

### Recombinant MyD88 TIR Protein

A synthetic DNA fragment corresponding to the cDNA encoding human MyD88 TIR domain residues 150–296 with BamH/Xhol restriction enzymes was from IDT (Integrated DNA technologies, IA, USA). DNA was amplified with the Ready-mix PCR kit (Sigma), and ligated (New England BioLabs, MA, USA) with the restriction enzymes BamH and Xhol (TaKaRa, Kusatsu, Shiga Prefecture, Japan) to pETM-11 SUMO3GFP fusion vector for protein expression in E. coli, a gift from the EMBL protein expression and purification core facility. Plasmids were gel purified with PureLink Quick Gel Extraction kit (Invitrogen, San Diego, CA, USA), sequence verified, and transformed to (C2987) NEB Dh5a competent cells (New England BioLabs) and amplified with a QIAGEN plasmid midiprep kit. The recombinant fusion protein- SUMO-MyD88-TIR was expressed in E. coli BL21 C43 (DE3) cells (Lucigen, WI, USA). *E. coli* BL21 C43(DE3) were grown in 2xYT medium containing 1% glucose, 1 M MgSO_4_ and 20xNPS (100 mM PO_4_, 25mMSO_4_, 50 mM NH_4_. 100 mM Na, 50 mM K) up to mid-log phase (O.D-0.6) in 37 °C and then were grown o.n at 25 °C with 0.4 mM IPTG (Sigma). The cells were lysed by centrifugation at 4 °C 10,000 RPM and then lysis buffer (Tris 50 mM pH 7.5, 10% glycerol, 0.5 M NaCl with 0.1% dodecyl maltoside, 1 mM PMSF, 0.2 mg/ml lysosome and 50 µg DNAse) was added to the cell pellets for 25 min on ice and cells were re-centrifuged at 4 °C 15,000 RPM for 15 min. The fusion protein was purified from the lysate with HIS-select Nickel Magnetic agarose beads (Sigma), and elution was performed with 300 mM imidazole (Sigma). In parallel, an identical procedure was performed to produce recombinant SUMO3-GFP fusion protein that served as a control.

### Competitive binding assay

The recombinant SUMO-MyD88-TIR protein (8 µg/ml) was attached to polystyrene microtiter 96 well plates (Nunc, Roskilde, Denmark) at 4 °C o.n. Wells were blocked with 1% Bovine Serum Albumin (Millipore, MA, USA) in PBS for one hour. 125 µM Biotinylated RDVLPGT (Biotin-MyDI, from Bio Basic Inc. Ontario, Canada) together with different concentrations (10, 1, 0.1, 0 µM) of *c*(MyD 4-4), MyDI or MyDI-sc were added for 30 min. After washing, Streptavidin-HRP (Biolegend, San Diego, CA, USA) was added for another 30 min. Wells were washed again and TMB substrate solution (Southern Biotech, AL, USA) was added for 5 min in the dark. After 5 min, 2 N sulfuric acid stop solution was added and absorbance was read at 450 nM in an Infinite 200 Pro plate reader (Tecan).

### Immunoprecipitation and Western blot

Plasmid pCMV-HA-MyD88 (full length) was a gift from Bruce Beutler (Addgene plasmid # 12287)^63^ and plasmid pCMV-Flag-1 MyD88 (full length) was obtained from Dundee university (Dundee, U.K). Plasmids were co-transfected by TurboFect reagent (ThermoFisher Scientific, MA, USA) to Human embryonic kidney (HEK) 293 T cells. After 48 hr MyD88 inhibitors or controls were added for three hr and in the last 20 minutes cells were incubated with 30 ng/ml IL-1β (ProSpec, Rehovot, Israel) in order to enhance the co-immunoprecipitation signal. Fig. S3G shows that co-immunoprecipitation (co-IP) samples from cells treated with IL-1β produced stronger bands than from cells not untreated samples, as shown by others^64^. Cells were lysed in Ripa buffer in the presence of protease inhibitors and incubated on ice for 25 min. Western blot was used to determine expression of the transgenes prior to immunoprecipitation. In brief, whole cell lysates were separated by gel electrophoresis and transferred to nitrocellulose membranes. Membranes were blocked with 5% skim milk for 1 hr and HA was detected using polyclonal mouse anti-HA (Novus Biologicals, Littleton, CO) followed by goat anti-mouse IgG-HRP (R&D systems, Minneapolis, MN). Flag was detected using polyclonal rabbit anti-Flag (R&D systems, Minneapolis, MN) followed by goat anti-rabbit IgG-HRP (Abcam, Cambridge, UK). Membranes were exposed to chemiluminescent substrate in the presence of hydrogen peroxide, using the E-ECL- chemiluminescense detection kit (Biological Industries, Israel), and images were captured using a Bio-Rad imaging system (Bio-Rad, Hercules, CA). For immunoprecipitation, cell lysates were incubated with Pierce Anti-HA magnetic beads (ThermoFisher Scientific, MA, USA) for 30 minutes at room temperature with mixing. After washing the beads, the supernatant containing the target antigen was eluted with 0.1 M glycine pH 2 at room temperature on a rotator for 8 minutes. Beads were separated magnetically, and the supernatants were separated by gel electrophoresis and Western blotting as described above.

### Analysis of p65 nuclear translocation

Hela cells were incubated for 3 hours with 20 µM *c*(MyD 4-4) and then activated with 20 ng/ml IL-1β or 10 ng/ml TNFα (ProSpec)) for 30 min or 1 hr, respectively. Cells were then fixed (3.7% PFA in PBS for 10 min), permeabilized (0.25% Triton-X100), and blocked (2% BSA in TBS) at 4 °C for 16 h. Cells were then stained with 0.6 µg/ml rabbit anti-p65 in 2% BSA in TBS (Santa Cruz Biotechnology, Dallas, TX) followed by 0.5 µg/ml CY-3 goat anti-rabbit antibody (Jackson ImmunoResearch, Baltimore Pike, PA). Cytoplasmic vs. nuclear localization was analyzed by fluorescent microscopy (Nikon-Ti microscope)^65^.

### Mice

C57BL/6 (B6) mice were purchased from Harlan (Jerusalem, Israel). Female, 8–14 week-old mice were used in the experiments. The mice were housed in the AAALAC approved SPF unit of our university, and all experiments were approved by the Hebrew University-Hadassah Institutional Animal Care and Use Committee, and all experiments were performed in accordance with relevant guidelines and regulations.

### Induction of EAE and treatment

Mice were immunized s.c. in the flank with 100 µg MOG_35–55_ emulsified in CFA supplemented with 300 µg *M*. *tuberculosis* (Mt) H37RA (BD Difco, NJ, USA). Pertussis Toxin (PTX, List Biological Laboratories, CA, USA) was injected i.p. at the time of immunization and 48 h later. In some experiments, animals were sacrificed prior to onset of clinical symptoms in order to analyze the draining lymph node response. EAE was scored on a scale of 0–6: 0, no impairment; 1, limp tail; 2, limp tail and hind limb paresis; 3, ≥1 hind limb paralysis; 4, full hind limb and hind body paralysis; 5, hind body paralysis and front limb paresis; 6, death.

### Draining lymph node (DLN) cell activation

Mice immunized s.c. with 100 µg MOG_35–55_/CFA supplemented with 300 µg *M*. *tuberculosis* (Mt) H37RA (BD Difco), injected i.p. with Pertussis Toxin (PTX, List Biological Laboratories) and treated with *c*(MyD 4-4) or control were sacrificed eleven days after immunization, and single cell suspensions from the popliteal, inguinal and axillary LNs were prepared. Cells were cultured in 96 well plates (0.5 × 10^6^ per well) for 72 h with or without increasing concentrations of MOG_35–55_ peptide.

### Statistical analysis

The 2-Tailed t test was used for statistical evaluation of all the results except the two way analysis of variance “ANOVA” test that was used for the EAE model. Values are shown for data that reached a significance of P ≤ 0.05 (*), P ≤ 0.01 (**), P ≤ 0.005, (***), P ≤ 0.001 (****). Bars show mean and standard deviation (s.d.) and in Fig. 2a-b and Fig. 6c standard error of the mean (s.e.m.) (Prism v.5, GraphPad Software Inc. San Diego, USA).

## Electronic supplementary material

Supplementary Information
