# Supplementary material for: Development of a Novel Backbone Cyclic Peptide Inhibitor of the Innate Immune TLR/IL1R Signaling Protein MyD88
Source: Sci Rep. 2018 Jun 21;8:9476. doi: 10.1038/s41598-018-27773-8 (PMC6013495; doi:10.1038/s41598-018-27773-8)
Supplement: Supplementary file 1 — Supplementary Information [file 41598_2018_27773_MOESM1_ESM.docx]

**Supplementary Information**

**Development of a Novel Backbone Cyclic Peptide Inhibitor of the Innate Immune TLR/IL1R Signaling Protein MyD88**

Shira Dishon^1^, Adi Shumacher^2^, Joseph Fanous^2^, Alaa Talhami^3^, Ibrahim Kassis^4^, Dimitrios Karussis^4^, Chaim Gilon^3^, Amnon Hoffman^2^ and Gabriel Nussbaum^1*^

Figure S1. ***c*(MyD 4-4)**  **blocks macrophage activation better than *c*(MyD 6-6)**. (A) THP-1 cells were treated with *c*(MyD 4-4) or *c*(MyD 6-6) for 3 hr and then stimulated with the TLR2 ligand Pam3CSK4. Human (h) TNFα levels were determined by ELISA at 24 hr. Data are representative of three separate experiments.

**A**

Competitive binding assay:

1. Coat microtiter plate with recombinant MyD88 SUMO-TIR fusion protein
2. Add biotinylated MyDI alone or together with MyDI/ *c*(MyD 4-4) /MyDI-sc
3. Incubate with Streptavidin-HRP
4. Add TMB/peroxide (substrate)
5. Stop reaction by addition of 2N H_2_SO_4_

Figure S2. **Competitive binding assay.** (A) Steps of the competitive binding assay: 1. Recombinant SUMO-MyD88 TIR fusion protein is coated o.n. to an Elisa plate at 8µg/ml and then wells are washed and blocked with 0.1% BSA for 1hr. 2. Wells are repetitively washed and linear biotin-MyDI is added at decreasing concentrations for 30 min. 3. wells are washed and incubated with Streptavidin-HRP for 30 min. 4. TMB/peroxide (substrate) is added until color develops (approximately 5 min) after which the reaction is stopped by the addition of 2N H_2_SO_4_. (B) Concentration dependent binding of biotin-MyDI to the SUMO-TIR fusion protein. (C) Absence of binding of biotin-MyDI to SUMO-EGFP fusion protein.

Figure S3. **Full length gels.** Full length gels of cropped gels shown in the manuscript (A-E). (A-C) represent the gels in figure 4B, the cropped bands are indicated with black boxes. (D-E) represent the gels in figure 4C, the cropped bands are indicated with black boxes. (F) is a full-length gel of a repetition of the experiment shown in figure S4E, in order to show that the higher molecular weight bands in S4E do not appear consistently. In (G) HEK cells were co-transfected with MyD88-HA and MyD88-FLAG, and then treated with or without 20ng/ml IL-1β for 30 min prior to immunoprecipitation with anti-HA beads and immunoblotting with anti-FLAG.

Table S1. **List of cyclic peptides screened in this study**
